# Kidney disease in the elderly: biopsy based data from 14 renal centers in Poland

**DOI:** 10.1186/s12882-016-0410-8

**Published:** 2016-11-25

**Authors:** Agnieszka Perkowska-Ptasinska, Dominika Deborska-Materkowska, Artur Bartczak, Tomasz Stompor, Tomasz Liberek, Barbara Bullo-Piontecka, Anna Wasinska, Agnieszka Serwacka, Marian Klinger, Jolanta Chyl, Michal Kuriga, Robert Malecki, Krzysztof Marczewski, Bogdan Hryniewicz, Tadeusz Gregorczyk, Monika Wieliczko, Stanislaw Niemczyk, Olga Rostkowska, Leszek Paczek, Magdalena Durlik

**Affiliations:** 1 Department of Transplantology, Nephrology and Internal Medicine, Medical University of Warsaw, Transplantation Institute, Warsaw, 02-006 Poland; 2 Department of Pathology, The Medical Center of Postgraduate Education, Warsaw, 01-813 Poland; 3 Department of Nephrology, Hypertension and Internal Medicine, University of Warmia and Mazury in Olsztyn, Olsztyn, 10-561 Poland; 4 Department of Nephrology, Transplantology and Internal Medicine, Medical University of Gdansk, Gdansk, 80-211 Poland; 5 Department of Internal Diseases, Nephrology and Transplantology, Central Clinical Hospital of the Ministry of Interior, Warsaw, 02-507 Poland; 6 Department of Nephrology and Transplantation Medicine, Wroclaw Medical University, Wroclaw, 50-556 Poland; 7 Dialysis Unit, Specialist Hospital in Radom, Radom, 26-600 Poland; 8 Department of Nephrology, District Hospital in Plock, Plock, 09-400 Poland; 9 Department of Internal Medicine and Nephrology, Specialist Hospital in Miedzylesie, Warszawa, 04-749 Poland; 10 Department of Nephrology, Endocrinology, Hypertension and Internal Diseases, Public District Hospital in Zamosc, Zamosc, 22-410 Poland; 11 Department of Nephrology and Dialysis, District Specialist Hospital in Slupsk, Slupsk, 76-200 Poland; 12 Department of Nephrology, District Hospital in Kielce, Kielce, 75-736 Poland; 13 Department of Nephrology, Dialysis, and Internal Diseases, Medical University of Warsaw, Warsaw, 02-097 Poland; 14 Department of Internal Diseases, Nephrology and Dialysis, Military Institute of Medicine, Warsaw, 04-141 Poland; 15 Department of Immunology, Transplantology and Internal Medicine, Medical University of Warsaw, Transplantation Institute, Warsaw, 02-006 Poland

**Keywords:** Kidney disease in elderly, Kidney biopsy, Glomerulonephritis, Hypertensive nephropathy

## Abstract

**Background:**

Longer life expectancy is associated with an increasing prevalence of kidney disease. Aging itself may cause renal damage, but the spectrum of kidney disorders that affect elderly patients is diverse. Few studies, mostly form US, Asia and West Europe found differences in the prevalence of some types of kidney diseases between elderly and younger patients based on renal biopsy findings, with varied proportion between glomerulopathies and arterionephrosclerosis as a dominant injury found. Here, for the first time in Eastern Europe we analyzed native kidney biopsy findings and their relationship to clinical characteristics at the time of biopsy in elderly individuals (aged ≥65) in comparison to younger adults (aged 18–64).

**Methods:**

Biopsy and clinical data from 352 patients aged ≥65 were retrospectively identified, analyzed and compared with a control group of 2214 individuals aged 18–64. All kidney biopsies studied were examined at Medical University of Warsaw in years 2009–14.

**Results:**

In elderly patients the leading indication for biopsy was nephrotic range proteinuria without hematuria (34.2%) and the most prevalent pathologic diagnoses were: membranous glomerulonephritis (MGN) (18.2%), focal segmental glomerulosclerosis (FSGS) (17.3%) amyloidosis (13.9%) and pauci immune glomerulonephritis (12.8%). Hypertension and age-related lesions very rarely were found an exclusive or dominant finding in a kidney biopsy (1.7%) and a cause of proteinuria (1.1%) in elderly individuals. There were 18.2% diabetics among elderly individuals, and as much as 75% of them had no morphologic signs of diabetic kidney disease in the renal biopsy. Amyloidosis, MGN, pauci immune GN, crescentic GN and light and/or heavy chain deposition disease (LCDD/HCDD) were more frequent whereas IgA nephropathy (IgAN), lupus nephritis (LN) and thin basement membrane disease (TBMD) were less common among elderly than in younger patients.

**Conclusions:**

Proteinuria, a dominating manifestation in elderly patients subjected to kidney biopsy was most commonly related to glomerulopathies. The relatively high prevalence of potentially curative kidney diseases in elderly individuals implicates the importance of renal biopsy in these patients.

**Electronic supplementary material:**

The online version of this article (doi:10.1186/s12882-016-0410-8) contains supplementary material, which is available to authorized users.

## Background

Elderly patients constitute the largest age group among all individuals with chronic kidney disease (CKD). The elderly are affected by the same types of kidney diseases as younger individuals, but their clinical course and morphological manifestation may be influenced by aging. Senescence is associated with an eGFR decline by approximately 0.8–1.7 ml/min per year, which limits renal function reserve and makes an individual more vulnerable to the influence of injurious factors, common in the elderly population, such as hypertension, cardiovascular disease, diabetes, and drugs nephrotoxicity [1, 2]. The complexity of the background as well as clinical and morphological manifestations of kidney injury impede precise disease recognition, make it difficult to establish prognosis, and hinder proper treatment selection. In a vast majority of cases renal biopsy is irreplaceable in identifying treatable, reversible lesions, as well as in defining both the activity and chronicity of kidney injury. It has been documented that the percentage of patients with the precise kidney disease recognition decreases with age and most of patients aged ≥55 are labelled as ‘CKD of unknown origin’ or ‘nephroangiosclerosis’ [3]. Many nephrologists no longer consider older age as a contraindication to immunosuppressive treatment, which is in line with the growing number of kidney biopsies performed in elderly patients [4].

## Methods

### Study design

This is a quantitative, descriptive and cross-sectional study that included a group of 2566 adult patients with kidney biopsies that were considered diagnostic (i.e., containing sufficient amount of renal cortex for light microscopic and immunomorphological evaluation as well as ultrastructural study whenever necessary for establishing the diagnosis), and were processed and interpreted over the period of 2009–2014 in the Nephropathological Laboratory, Department of Transplantology, Nephrology and Internal Medicine at Medical University of Warsaw. Among patients studied we identified 352 elderly individuals (accepting an defining age of ≥65 [5]) as well as a control group of 2214 patients aged 18–64 years. We analyzed kidney-biopsy based diagnoses in terms of their prevalence and clinicopathological associations in both cohorts. The list of clinical data subjected to analysis included patients’ gender, age, eGFR (MDRD), a pre-biopsy follow-up duration, the presence of diabetes, hypertension, and urinalysis results. The clinical categories of renal disease at the time of biopsy were defined as follows: nephrotic range proteinuria (≥3.5 g/day) with or without hematuria, non-nephrotic proteinuria (<3.5 g/day) with or without hematuria, isolated hematuria.

To base our study on most credible and objective measurements, all presumptive and interpretative data, such as those relating to the acuteness/chronicity as well as dynamics of kidney disease evolution, were excluded from the analysis. As the definition of Acute Kidney Injury (AKI) has been changing over the last several years and we were not able to verify the historical data, we didn’t include this clinical course characteristic in our analysis.

### Pathologic diagnoses

In all cases light microscopic (LM) evaluation and immunofluorescence (IFL) (for IgG, IgA, IgM, C3, C1q, fibrinogen, kappa and lambda lights chains) were performed. The diagnosis of immune-complex mediated glomerulopathies, such as IgAN, MGN, LN, etc. was based on the IFL and LM findings. In 44.8% of biopsies an examination in electron microscopy was also done, which was decided whenever the final diagnosis could not have been made without it. Most commonly the ultrastructural analysis was performed due to: 1) an unspecific/incoherent LM and/or IFL findings; 2) a suspicion of different nephropathies overlap (e.g., DKD coexisting with immune-complex mediated glomerulonephritis); 3) a discrepancy between clinical patients’ characteristics and morphological findings in LM and IFL; and 4) a suspected paraproteinemia. The list of morphological diagnoses is presented in Additional file 1: Table S1. We decided not to use the distinction between primary and secondary nephropathies assuming that at least some cases considered primary at the time of biopsy might have later changed their category once a detailed diagnostic process was completed.

### Statistical analyses

The statistical analysis was performed using SAS 9.4 software for Windows. Quantitative variables were summarized by medians (ranges), because the parameters did not follow a normal distribution and they were compared using Wilcoxon Rank-Sum test. Qualitative variables were compared using the *χ*2-test and Fisher’s exact test, respectively to the sample size. A *P*-value of <0.05 was considered statistically significant.

## Results

**Fig. 1:**
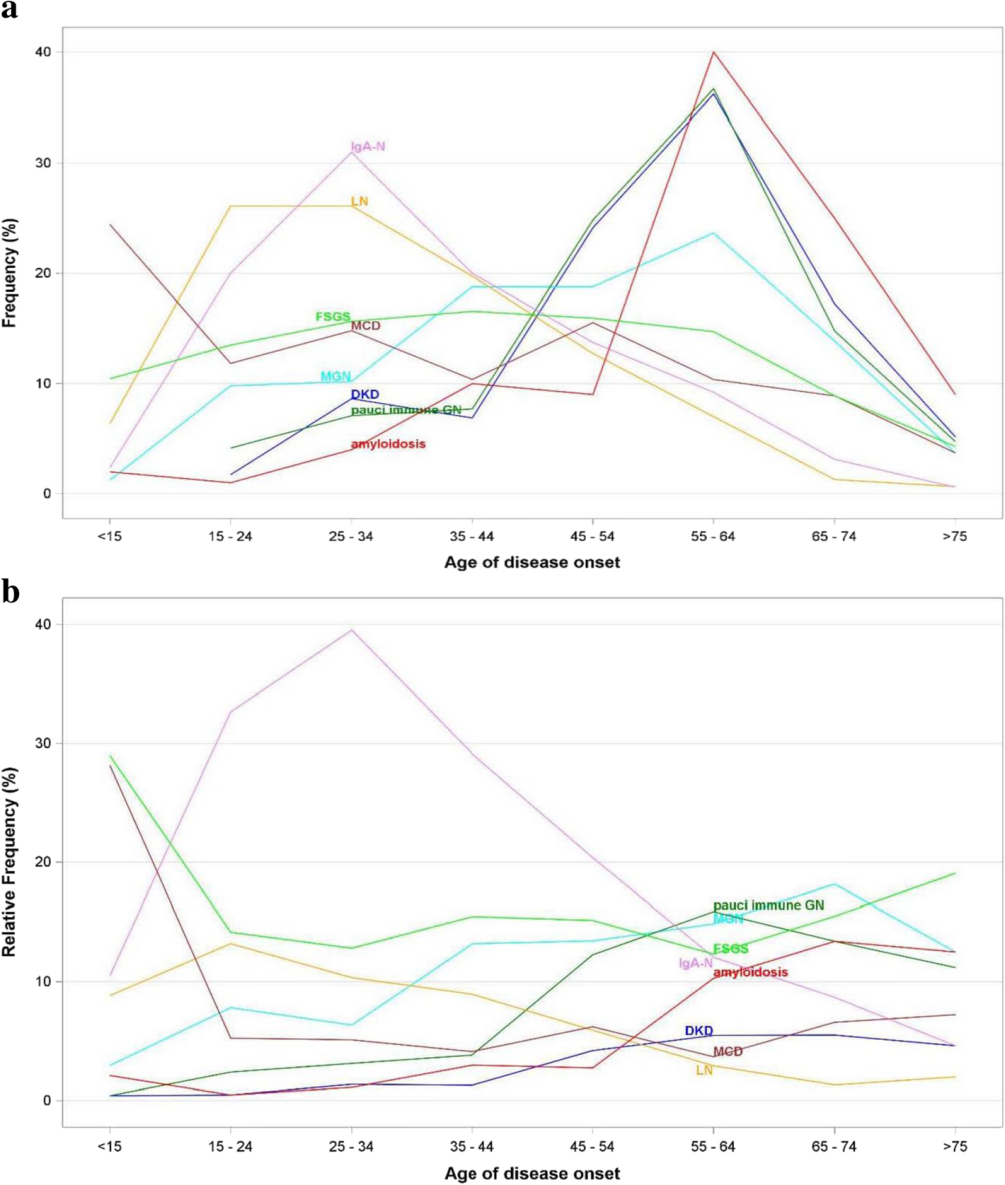
Frequency (**a**) and relative frequency (**b**) distribution of selected kidney biopsy based diagnoses according to age of disease onset

Baseline characteristics are presented in Table 1. The spectrum of renal biopsy diagnoses in elderly and younger patients is shown in Table 2. Three leading renal biopsy diagnoses among elderly patients were MGN, FSGS, and amyloidosis, whereas in younger individuals IgAN, FSGS and MGN were most frequent ones. In comparison to younger patients the elderly cohort was characterized by higher prevalence of MGN, amyloidosis, *pauci immune* GN, crescentic GN, tubulointerstitial inflammation, and light chain/heavy chain deposition disease (LCDD/HCDD) as well as lower prevalence of IgAN, lupus nephritis (LN) and thin basement membrane disease (TBMD). The pre-biopsy follow-up data (Additional file 2: Table S2) have been used to define patients’ age at the disease onset, and in the frequency analysis of selected nephropathies. Figure 1a illustrates the frequency of selected nephropathies at different age-groups, whereas Fig. 1b demonstrates their frequencies in relation to all nephropathies (renal biopsy based diagnoses) occurring in a particular age group.

**Table 1:** Clinical characteristics at the time of biopsy by age

	Elderly (aged ≥65) (*n* = 352)	Younger (aged 18–64) (*n* = 2214)	*P*
Male/female ratio	1:1	1.1/1	
Age of disease onset	median: 69 (range: 19–87)	median: 37 (range: 1–64)	<0.001
Pre-biopsy follow-up (months)	median: 5 (range: 0–660)	median: 10 (range: 0–612)	<0.001
Pre-biopsy follow-up			
<3 months > 3 months	165 (46.9%) 187 (53.1%)	848 (38.3%) 1366 (61.7%)	0.002
Proteinuria			
nephrotic non-nephrotic	188 (55.6%) 134 (39.6%)	916 (42.8%) 1124 (52.5%)	<0.001
Hematuria	159 (45.3%)	1359 (61.8%)	<0.001
Nephrotic range proteinuria without hematuria	115 (34.2%)	453 (21.3%)	<0.001
Non-nephrotic proteinuria with hematuria	76 (22.5%)	787 (37%)	
Nephrotic range proteinuria with hematuria	73 (21.6%)	461 (21.6%)	
Non-nephrotic proteinuria without hematuria	58 (17.2%)	328 (15.4%)	
Isolated hematuria	8 (2.4%)	72 (3.4%)	
eGFR mL/min/1.73 m^2^	median 39.3 (range 2.5–161.6)	median 66.8 (range 2.7–252)	<0.001
eGFR ≥ 60 mL/min/1.73 m^2^	92 (26.1%)	1194 (53.9%)	<0.001
eGFR 30–59 mL/min/1.73 m^2^	113 (32.1%)	548 (24.8%)	
eGFR 15–29 mL/min/1.73 m^2^	73 (20.7%)	238 (10.8%)	
eGFR < 15 mL/min/1.73 m^2^	74 (21%)	234 (10.5%)	
Duration of symptoms < 3 months	165 (46.9%)	847 (38.3%)	0.002
Duration of symptoms ≥3 months	187 (53.1%)	1367 (61.7%)	
Hypertension	287 (84.2%)	1398 (67.1%)	<0.001
Diabetes	64 (18.5%)	160 (7.5%)	<0.001
Steroid treatment before the biopsy	59 (17.5%)	491 (23.7%)	0.012
Other immunosuppressive treatment during pre-biopsy follow-up	16 (4.8%)	187 (9.0%)	0.008

**Table 2:** Renal biopsy diagnoses by age

Diagnosis	Elderly (aged ≥65) (*n* = 352)	Younger (aged 18–64) (*n* = 2214)	*P*
MGN	64 (18.2%)	227 (10.3%)	<0.001
FSGS	61 (17.3%)	339 (15.3%)	0.342
Amyloidosis AL AA Non AA, non AL /not defined	49 (13.9%) 10 (2.8%) 25 (7.1%) 14 (4%)	82 (3.7%) 24 (1.1%) 39 (1.8%) 19 (0.8%)	<0.001 0.019 <0.001 <0.001
*Pauci immune* GN crescentic GN focal segmental GN	45 (12.8%) 26 (7.4%) 19 (5.4%)	150 (6.8%) 82 (3.7%) 68 (3%)	<0.001 0.004 0.037
IgAN	33 (9.4%)	604 (27.3%)	<0.001
Crescentic GN (types I,II,III)	31 (8.8%)	109 (4.9%)	0.005
MCD	22 (6.3%)	132 (6%)	0.809
DKD	16 (4.6%)	62 (2.9%)	0.100
Unclassified lesions	14 (4%)	92 (4.2%)	1.000
Tubulointerstitial nephritis	9 (2.6%)	34 (1.5%)	0.177
Membrano-proliferative GN	8 (2.3%)	67 (3%)	0.608
Arterionephrosclerosis (hypertensive nephropathy and aging nephropathy)	6 (1.7%)	18 (0.8%)	0.128
MGA	6 (1.7%)	79 (3.6%)	0.077
End-stage kidney	6 (1.7%)	39 (1.8%)	1.000
LN	5 (1.4%)	191 (8.6%)	<0.001
LCDD/HCDD	4 (1.1%)	6 (0.3%)	0.037
Acute tubular injury/necrosis	4 (1.1%)	8 (0.4%)	0.070
Thrombotic microangiopathy	3 (0.9%)	39 (1.8%)	0.263
Normal kidney morphology	2 (0.6%)	29 (1.3%)	0.303
TBMD	1 (0.3%)	40 (1.8%)	0.036

The table encompasses only the diagnoses which were made in at least 1% of individuals in any of the two cohorts studied

### Clinical manifestation of kidney disease in elderly and younger patients

A majority among elderly patients presented nephrotic range proteinuria (55.6%) in contrast to younger cohort in which non-nephrotic proteinuria dominated (52.5%, *P* < 0.001) (Table 1). Among elderly patients with nephrotic range proteinuria the leading diagnoses were MGN (25%), amyloidosis (20.2%) and FSGS (20.2%) in contrast to younger cohort with this clinical syndrome, in which most prevalent were FSGS (17.5%), MGN (16.9%) and IgAN (16.8%). Among elderly patients, a majority of those with nephrotic range proteinuria had no hematuria, and this clinical presentation was most commonly associated with amyloidosis (25.2%) (Table 3).

**Table 3:** Distribution of renal biopsy diagnoses among elderly and younger adult patients with nephrotic range proteinuria

	Nephrotic range proteinuria with hematuria		*P*	Nephrotic range proteinuria without hematuria		*P*	Nephrotic range proteinuria with or without hematuria		*P*
Renal pathology	≥65 (*n* = 73)	18–64 (*n* = 461)		≥65 (*n* = 115)	18–64 (*n* = 453)		≥65 (*n* = 188)	18–64 (*n* = 916)	
MGN	22 (30.1%)	49 (10.6%)	<0.001	25 (21.7%)	106 (23.4%)	0.804	47 (25.0%)	155 (16.9%)	0.013
FSGS	15 (20.6%)	79 (17.1%)	0.508	23 (20.0%)	81 (17.9%)	0.591	38 (20.2%)	160 (17.5%)	0.404
Amyloidosis	9 (12.3%)	13 (2.8%)	0.001	29 (25.2%)	44 (9.7%)	<0.001	38 (20.2%)	57 (6.2%)	<0.001
*Pauci immune* GN crescentic GN focal segmental GN	7 (9.6%) 3 (4.1%) 4 (5.5%)	31 (6.7%) 18 (3.9%) 13 (2.8)	0.336 1.000 0.271	3 (2.6%) 1 (0.9%) 2 (1.7%)	4 (0.9%) 2 (0.4%) 2 (0.4%)	0.151 0.493 0.184	10 (5.3%) 4 (2.1%) 6 (3.2%)	35 (3.8%) 20 (2.2%) 15 (1.6%)	0.317 1.000 0.151
IgAN	6 (8.2%)	114 (24.7%)	0.001	4 (3.5%)	39 (8.6%)	0.075	10 (5.3%)	154 (16.8%)	<0.001
MCD	5 (6.9%)	30 (6.5%)	0.804	11 (9.6%)	84 (18.5%)	0.025	16 (8.5%)	114 (12.5%)	0.137
Membrano-proliferative GN	5 (6.9%)	30 (6.5%)	0.804	1 (0.9%)	11 (2.4%)	0.475	6 (3.2%)	41 (4.5%)	0.553
DKD	2 (2.7%)	26 (5.6%)	0.405	13 (11.3%)	23 (5.1%)	0.029	15 (8.0%)	49 (5.4%)	0.170
LN	1 (1.4%)	51 (11.1%)	0.005	0	23 (5.1%)	0.007	1 (0.5%)	74 (8.1%)	<0.001
End-stage kidney	1 (1.4%)	4 (0.9%)	0.522	1 (0.9%)	8 (1.8%)	0.695	2 (1.1%)	12 (1.3%)	1.000
Arterionephrosclerosis	1 (1.4%)	2 (0.4%)	0.357	1 (0.9%)	5 (1.1%)	1.000	2 (1.1%)	7 (0.8%)	0.655
MGA	0	3 (0.7%)	1.000	1 (0.9%)	11 (2.4%)	0.475	1 (0.5%)	15 (1.6%)	0.498
Thrombotic microangiopathy	0	9 (2.0%)	0.618	1 (0.9%)	6 (1.3%)	1.000	1 (0.5%)	15 (1.6%)	0.498
Unclassified lesions	0	13 (2.8%)	0.232	3 (2.6%)	14 (3.1%)	1.000	3 (1.6%)	27 (3.0%)	0.458

Among elderly patients with non-nephrotic range proteinuria the most common diagnoses were *pauci immune* GN (21.6%), FSGS (17.2%) and IgAN (14.9%), whereas in individuals aged 18–64 years with this clinical syndrome IgAN (36.7%), FSGS (14.5%) and LN (9.1%) were most prevalent ones (Table 4).

**Table 4:** Distribution of renal biopsy diagnoses among elderly and younger patients with non-nephrotic range proteinuria

	Non-nephrotic range proteinuria with hematuria		*P*	Non-nephrotic range proteinuria without hematuria		*P*	Non-nephrotic range proteinuria with or without hematuria		*P*
Renal pathology	≥65 (*n* = 76)	18–64 (*n* = 787)		≥65 (*n* = 58)	18–64 (*n* = 328)		≥65 (*n* = 134)	18–64 (*n* = 1124)	
*Pauci immune* GN crescentic GN focal segmental GN	25 (32.9%) 15 (19.7%) 10 (13.2%)	92 (11.7%) 52 (6.6%) 40 (5.1%)	<0.001 <0.001 0.009	4 (6.9%) 2 (3.5%) 2 (3.5%)	7 (2.1%) 2 (0.6%) 5 (1.5%)	0.067 0.109 0.284	29 (21.6%) 17 (12.7%) 12 (9.0%)	99 (8.8%) 54 (4.8%) 45 (4.0%)	<0.001 0.001 0.015
IgAN	15 (19.7%)	336 (42.7%)	<0.001	5 (8.6%)	74 (22.6%)	0.013	20 (14.9%)	413 (36.7%)	<0.001
FSGS	11 (14.5%)	97 (12.3%)	0.586	12 (20.7%)	65 (19.8%)	0.860	23 (17.2%)	163 (14.5%)	0.439
Unclassified lesions	7 (9.2%)	29 (3.7%)	0.032	3 (5.2%)	24 (7.3%)	0.781	10 (7.5%)	53 (4.7%)	0.204
MGN	4 (5.3%)	27 (3.4%)	0.342	10 (17.2%)	40 (12.2%)	0.292	14 (10.5%)	67 (6.0%)	0.046
LN	2 (2.6%)	73 (9.3%)	0.049	2 (3.5%)	28 (8.5%)	0.285	4 (3.0%)	102 (9.1%)	0.013
Tubulointerstitial nephritis	2 (2.6%)	4 (0.5%)	0.091	4 (6.9%)	10 (3.1%)	0.242	6 (4.5%)	16 (1.4%)	0.023
LCDD/HCDD	2 (2.6%)	2 (0.3%)	0.041	1 (1.7%)	1 (0.3%)	0.278	3 (2.2%)	3 (0.3%)	0.019
Membrano-proliferative GN	2 (2.6%)	15 (1.9%)	0.656	0	9 (2.7%)	0.366	2 (1.5%)	24 (2.1%)	1.000
End-stage kidney	2 (2.6%)	18 (2.3%)	0.694	2 (3.5%)	7 (2.1%)	0.630	4 (3%)	25 (2.2%)	0.540
TBMD	1 (1.3%)	23 (2.9%)	0.715	0	2 (0.6%)	1.000	1 (0.8%)	25 (2.2%)	0.513
MCD	1 (1.3%)	5 (0.6%)	0.426	4 (6.9%)	8 (2.4%)	0.089	5 (3.7%)	13 (1.2%)	0.035
Amyloidosis	1 (1.3%)	7 (0.9%)	0.523	8 (13.8%)	15 (4.6%)	0.013	9 (6.7%)	23 (2.1%)	0.005
MGA	0	31 (3.9%)	0.102	1 (1.7%)	16 (4.9%)	0.281	1 (0.8%)	48 (4.3%)	0.046
DKD	0	8 (1.0%)	1.000	1 (1.7%)	5 (1.5%)	1.000	1 (0.8%)	13 (1.2%)	1.000
Thrombotic microangiopathy	0	10 (1.3%)	1.000	1 (1.7%)	9 (2.7%)	1.000	1 (0.8%)	20 (1.8%)	0.718
Arterionephrosclerosis	0	2 (0.3%)	1.000	2 (3.5%)	6 (1.8%)	0.344	2 (1.5%)	8 (0.7%)	0.289
Normal kidney morphology	0	12 (1.5%)	0.614	0	2 (0.6%)	1.000	0	14 (1.3%)	0.385
Alport syndrome	0	8 (1.0%)	1.000	0	2 (0.6%)	1.000	0	10 (0.9%)	1.000

An isolated hematuria was seen in only 2.4% of elderly patients studied, and was most commonly associated with nonspecific lesions best defined as ‘minor glomerular abnormalities’ (MGA). In younger individuals an isolated hematuria was a presenting symptom in only 3.4% of patients aged <65, and was most commonly associated with IgA-N (23.6%), MGA (18.1%), and TBMD (14.0%).

### Renal biopsy diagnoses according to gender in the elderly

Significant discrepancies between sexes were found in the frequency of amyloidosis AA, more commonly seen in females (10.8% vs. 3.4%, *P* = 0.011) and IgA-N, significantly more frequently recognized in males (13.1% vs. 5.7%, *P* = 0.027).

### Types of kidney disease in elderly diabetic individuals

Among patients aged ≥65 there were 64 (18.2%) individuals with diabetes. The median time of diabetes duration at the time of kidney biopsy was 7.16 years (range 0–24 years). In 11 (17%) of these patients kidney biopsy revealed lesions consistent with pure DKD, in 4 (6%) patients there were morphological features of DKD coexisting with another type of kidney injury, and in 48 (75%) elderly diabetic patients there were no microscopic features of DKD (Table 5). Among elderly patients with diabetes and no DKD on kidney biopsy the most prevalent type of kidney disease was *pauci immune* GN (focal segmental and crescentic) associated with the presence of ANCA.

**Table 5:** Renal biopsy diagnoses among patients with diabetes

Patients with diabetes	Kidney biopsy diagnosis			
	Diabetic kidney disease absent	Diabetic kidney disease present	Coincidence of diabetic kidney disease and other nephropathy	*P*
≥ 65 (*n* = 64)	48 (75.0%)	11 (17.0%)	4 (6.0%)^a^	0.612
18–64 (*n* = 160)	98 (61.3%)	52 (32.5%)	10 (6.3%)^b^	

^a^2 cases of MGN, 1case of MCD and 1 case of IgAN

^b^6 cases of IgA-N, 1 case of LCDD, 1case of MCD, 2 cases of MGN

## Discussion

CKD, together with diabetes and cardiovascular disease constitute three interrelated conditions of strong public health relevance [6]. Reduced estimated eGFR is one of the very important risk factors of cardiovascular disease and death [7]. In US about 30–40% of adults aged ≥70, and approximately 50% of those aged ≥80 have CKD [8, 9]. In Poland the prevalence of CKD in the elderly population has been recently estimated at 29.4% [10]. In the elderly CKD exerts stronger effect on the life expectancy than in younger population [11]. Therefore, early detection of kidney disease, its type recognition, and the implementation of targeted treatment should be regarded as tools necessary to limit CKD complications and to improve outcomes in cardiovascular diseases.

The complexity of renal injury among elderly individuals obscures the clinical picture. Without a renal biopsy the establishment of the proper diagnosis may be impossible, or at least protracted [12–14].

There are no strict criteria qualifying patients to kidney biopsy in any age group. As it has been defined by Bomback et al., kidney biopsy should in general be considered in any patient who presents at least 2 of the following findings: hematuria, proteinuria ≥1 g/day, renal insufficiency, and/or positive serologies for systemic diseases with known potential for kidney involvement (e.g., hepatitis B or C virus infection, systemic lupus erythematosus and ANCA seropositivity) [15]. As it was emphasized, these indications apply to all age-groups, including elderly (aged ≥65) and very elderly (aged ≥80 years) patients. Several studies confirmed that age is not a significant risk factor for biopsy-related complications [13, 16, 17].

Although kidney biopsy offers the deepest insight into the renal tissue injury, its morphological interpretation may be problematic in the case of significant interference by lesions related to aging, such as global glomerulosclerosis, arteriosclerosis, arteriolar hyalinization, interstitial fibrosis and tubular atrophy. The additional background injury may also be caused by long-lasting hypertension and/or diabetes, as well as several environmental and iatrogenic factors, which are relatively common in the elderly population.

In our elderly patients studied the most common clinical manifestation of renal disease was nephrotic syndrome (55.6%) with or without hematuria (21.6 and 34.2%, respectively) which was consistent with the observations of others [14, 16, 18–21]. There are also reports documenting AKI as a leading indication for native kidney biopsies in the elderly with nephrotic syndrome being the second most common manifestation of renal disease in this patient group [14, 22]. What seems to be a problem is the difficulty to define the epidemiology of AKI. This is partly due to the fact that the clinical picture of AKI is very heterogeneous, which has been reflected by the existence of various classification systems based on different diagnostic criteria with the most recent one announced by KDIGO in 2012 [23]. Our study covered the period between 2009 and 2014, the clinical data were provided by different renal centers, and the descriptions of a disease course were not uniform and precise enough to allow for a reliable distinction between AKI, AKI superimposed on CKD and ‘pure’ CKD.

Although the spectrum of diseases affecting people aged ≥65 is the same as in younger population, there are some distinct differences in the frequency of certain nephropathies between these two age groups. Our observation of the relatively high incidence of MGN among elderly individuals is in line with the reports of others [12, 13, 22, 24, 25]. Our data indicate that the peak frequency of MGN occurs at the age 55-64 years, and a peak relative frequency at the age of 65–74 years (Fig. 1). The histological recognition of MGN provides important rationale for the use of immunosuppressive therapy, especially if progressive eGFR lowering is observed.

Focal segmental glomerulosclerosis was recognized as the second most common histological diagnosis in both elderly and younger patients (17.3 and 15.3% respectively). As in our study, an association between FSGS and nephrotic-range proteinuria, as well as no difference in FSGS frequency between patients aged ≥65 and those aged <65 were observed by others [13, 16, 17]. In a vast majority of our cases studied morphological and clinical features suggested the secondary nature of glomerular sclerosis, but the etiology of this process was ambiguous. Although in many elderly patients a contribution of aging, hypertension and arteriosclerosis to the FSGS development could not be excluded, the histological picture was not specific enough to justify a diagnosis of hypertensive nephropathy or arterionephrosclerosis.

In a few published studies that analyzed the prevalence of kidney biopsy based diagnoses in elderly individuals the percentage of cases, in which age and/or hypertension related lesions were dominant microscopic finding ranged from 1.6% in Chinese patients to 6.2% in Japanese cohort [19, 24, 26]. The proportion of aforementioned diagnosis in our elderly group was 1.7% (Table 2), despite the fact that as much as 84.2% of patients aged ≥65 suffered from hypertension (Table 1.)

Amyloidosis was found to be the third most common histological diagnosis in our elderly patients with a prevalence of 13.9% as compared with 3.7% in younger individuals (*P* < 0.001). These findings are in line with the reports of others [13, 17, 20]. Our data show the peak frequency of amyloidosis at the age of 55–64, whereas the highest relative frequency of this disease occurred at the age of 65–74 (Fig. 1). Amyloidosis was found to be the leading (25.2%) histological finding among elderly patients with nephrotic range proteinuria without hematuria (Table 3.). Although a nephrotic-range proteinuria without hematuria was the most common presentation of amyloidosis in the elderly (61.7%), in a substantial percentage of cases (19%) the disease was manifested by non-nephrotic proteinuria. The latter finding should be emphasized, since many nephrologists refrain from kidney biopsy in an elderly patient with mild proteinuria, in whom concomitant amyloidosis-related cardiomyopathy can be easily misinterpreted as an ischemic heart disease.

Several authors reported the proportion of diabetic kidney disease ranging between 2.2% up to 10% in renal biopsy registries [19, 24]. The results of ours implicate that in people aged ≥65 DKD constitutes 4.6% of all renal-biopsy diagnoses. In our cohort the peak frequency and the highest relative frequency of this disease occurred at the age of 55–64 years (Fig. 1). The usual indications for renal biopsy in diabetic patients include: an abrupt onset or rapid progression of kidney disease, the presence of active urinary sediment, and the progression of proteinuria in the absence of diabetic retinopathy or neuropathy. Since the group of diabetic patients in our cohort is not representative for the whole population of diabetics, it should be noted that 75% of our elderly diabetic patients had non-diabetic kidney disease upon kidney biopsy, which is in line with the observation made by Sharma et al. [27]. These results may be partially explained by the relatively short duration of diabetes at the time of kidney biopsy in our patients (median 7.16 years for diabetic patients studied, range 0–24 years), as well as by the fact that in 40% of biopsies from diabetic individuals electron microscopy was not performed, which does not allow for the exclusion of early phase of DKD.

One of the leading histological diagnoses in general, and the most common renal disease manifested by non-nephrotic range proteinuria with hematuria in our elderly patients was *pauci-immune* GN. Notably, 23.8% of elderly and 24.8% of younger patients with *pauci-immune* GN suffered from nephrotic-range proteinuria. *Pauci-immune* GN being one of the leading diagnoses made upon kidney biopsy among elderly individuals was reported by others [13, 25, 28, 29]. The peak frequency and the highest relative frequency of this disease in our patients studied occurred at the age of 55-64 years (Fig. 1). In a vast majority of cases *pauci immune* GN is associated with the presence of ANCA vasculitis, with peak incidence of 52.9 per million being observed among people aged 65–74 [30]. It is worth mentioning that in contrast to younger individuals, in the elderly vasculitis more commonly manifests with renal disease, and less frequently involves upper respiratory tract [29]. Vasculitis-related symptoms such as myalgia and headache may be easily misdiagnosed as common afflictions of the elderly, e.g., polymyalgia rheumatica [31].

## Conclusions

Our analysis confirmed differences in the prevalence of certain kidney diseases between elderly and younger individuals. Elderly patients were more frequently nephrotic, hypertensive and diabetic, and have lower eGFR at the time of the biopsy than younger individuals. A leading clinical manifestation in patients aged ≥65 years submitted to kidney biopsy was proteinuria, most commonly of nephrotic range, which in majority of cases was related to glomerulopathies, Hypertension and age-related lesions rarely were found an exclusive or dominant finding in a kidney biopsy or a cause of proteinuria in individuals aged ≥65 years. The relatively high prevalence of potentially curative kidney diseases in the elderly individuals implicates the importance of renal biopsy in these patients.

## Additional files

Additional file 1: Table S1. The list of kidney biopsy diagnoses. Contains morphological diagnoses which occurred at least once in the study group. (DOC 33 kb)
Additional file 2: Table S2. Pre-biopsy follow-up in selected nephropathies in elderly and younger patients. (DOCX 12 kb)

## Abbreviations

AKI: Acute kidney injury
CKD: Chronic kidney disease
DKD: Diabetic kidney disease
FSGS: Focal segmental glomerulosclerosis
GN: Glomerulonephritis
IFL: Immunofluorescence
IgAN: IgA nephropathy
LCDD/HCDD: Light and/or heavy chain deposition disease
LM: Light microscopy
LN: Lupus nephritis
MCD: Minimal change disease
MGA: Minor glomerular abnormalities
MGN: Membranous glomerulonephritis
TBMD: Thin basement membrane disease
