# Supplementary material for: Kidney disease in the elderly: biopsy based data from 14 renal centers in Poland
Source: BMC Nephrol. 2016 Nov 25;17:194. doi: 10.1186/s12882-016-0410-8 (PMC5123353; doi:10.1186/s12882-016-0410-8)
Supplement: Additional file 1: Table S1. — The list of kidney biopsy diagnoses. Contains morphological diagnoses which occurred at least once in the study group. (DOC 33 kb) [file 12882_2016_410_MOESM1_ESM.doc]

# **Kidney disease in the elderly: biopsy-based data from 14 renal centres in Poland**

Table S1. The list of kidney biopsy diagnoses*

| Type of nephropathy | Diagnoses |
| --- | --- |
| Glomerulopathy | Minor glomerular abnormalities (MGA), difficult to classify glomerulopathy, minimal change disease (MCD), IgM nephropathy, C1q nephropathy, focal segmental glomerulosclerosis (FSGS), nodular glomerulosclerosis, membranous glomerulonephritis (MGN), IgA nephropathy (IgA-N), diffuse endocapillary GN, acute postinfectious GN with humps, focal segmental GN (not IgAN, lupus GN, C1q GN, etc.), crescentic GN types I, II, III, membrano-proliferative GN (MPGN) types I, III, dense deposits disease, C3 GN, mesangioproliferative GN (not IgAN, lupus GN, C1q GN, etc.), Alport’s syndrome/hereditary nephritis with GBM lamination, thin basement membrane nephropathy, fibrillary GN, immunotactoid glomerulopathy, fibronectin glomerulopathy, Waldenstrom macroglobulinema, light and/or heavy chain deposition disease (LCDD/HCDD), collagenofibrotic nephropathy, amyloidosis, diabetic kidney disease (DKD), Fabry disease, LCAT deficiency, lipoprotein glomerulopathy, hepatic glomerulosclerosis, sickle cell glomerulopathy, lupus nephritis |
| Tubulointerstitial nephropathy (TIN) | Acute tubular injury, light chain tubulopathy, cast nephropathy, myoglobin/hemoglobin cast nephropathy, gout nephropathy, nephrocalcinosis, phosphate nephropathy, oxalosis, cystinosis, acute nondestructive tubulointerstitial nephritis (TIN), chronic nondestructive TIN, acute pyelonephritis, chronic pyelonephritis/reflux nephropathy, xanthogranulomatous or malacoplakia/megalocytic TIN, analgetic nephropathy, granulomatous TIN,  nephronophthisis/medullary cystic disease, IgG4 related disease |
| Vasculopathy | Thrombotic microangiopathy, arterionephrosclerosis (hypertensive nephrosclerosis), renal artery stenosis atrophy, atheroembolization (cholesterol embolization), calcineurin inhibitor toxicity, arteritis |
| Other | Normal kidney morphology,  end stage renal disease NOS |

* the table contains morphological diagnoses which occurred at least once in the study group.
