# Supplementary material for: Kidney disease in the elderly: biopsy based data from 14 renal centers in Poland
Source: BMC Nephrol. 2016 Nov 25;17:194. doi: 10.1186/s12882-016-0410-8 (PMC5123353; doi:10.1186/s12882-016-0410-8)
Supplement: Additional file 2: Table S2. — Pre-biopsy follow-up in selected nephropathies in elderly and younger patients. (DOCX 12 kb) [file 12882_2016_410_MOESM2_ESM.docx]

****Kidney disease in the elderly: biopsy-based data from 14 renal centres in Poland****

**Table S2. Pre-biopsy follow-up in selected nephropathies in elderly and younger patients**

|  | **Pre-biopsy follow-up (months)**  **median (range)** | | ***P*** |
| --- | --- | --- | --- |
| **Renal biopsy diagnosis** | **Elderly (aged ≥65)** | **Younger (aged 18-64)** |  |
| Membranous glomerulonephritis | 7.5 (1-660) | 7.0 (0-342) | 0.555 |
| Amyloidosis  AL  AA  non AA, non AL/ not defined | 9 (0-97)  9 (1-20)  8 (1-97)  10 (0-75) | 8 (0-445)  5 (1-48)  11 (0-233)  12 (1-445) | 0.254  0.892  0.365  0.254 |
| Focal segmental glomerulosclerosis | 4 (0-161) | 14 (0-612) | <0.001 |
| *Pauci immune* GN  crescentic GN  focal segmental GN | 2.5 (0-153)  2 (0-30)  3.5 (1-153) | 2 (0-130)  2 (0-130)  3 (0-81) | 0.345  0.714  0.401 |
| Minimal change disease | 2.5 (0-60) | 3 (0-380) | 0.100 |
| IgA nephropathy | 9 (1-382) | 18 (0-483) | 0.090 |
| Diabetic kidney disease | 3 (0-166) | 6 (1-210) | 0.267 |
| Lupus nephritis | 28 (2-54) | 40 (0-302) | 0.457 |
